# Clinical, radiographic and molecular characterization of two unrelated families with multicentric osteolysis, nodulosis, and arthropathy

**DOI:** 10.1186/s12891-023-06856-2

**Published:** 2023-09-14

**Authors:** Tayyaba Ishaq, Petra Loid, Hafiza Abida Ishaq, Go Hun Seo, Outi Mäkitie, Sadaf Naz

**Affiliations:** 1 School of Biological Sciences, University of the Punjab, Quaid-i-Azam Campus, Lahore, 54590 Pakistan; 2 Folkhälsan Research Center, Genetics Research Program, Helsinki, Finland; 3 Children’s Hospital, University of Helsinki and Helsinki University Hospital, Helsinki, Finland; 4 Department of Clinical Genetics, Helsinki University Hospital, Helsinki, Finland; 5 Research Program for Clinical and Molecular Metabolism, University of Helsinki, Helsinki, Finland; 6 Services Hospital Lahore, Lahore, Pakistan; 7 3billion, Inc, Seoul, Republic of Korea; 8 Department of Molecular Medicine and Surgery, Center for Molecular Medicine, Karolinska Institute, Stockholm, Sweden

**Keywords:** Exome sequencing, Pakistan, Finland, Skeletal deformities, Nodulosis, Arthropathy, Longitudinal survey, MMP2

## Abstract

**Background:**

Multicentric osteolysis nodulosis and arthropathy (MONA) is a rare autosomal recessive disorder characterized by marked progressive bone loss and joint destruction resulting in skeletal deformities. MONA is caused by MMP2 deficiency. Here we report clinical and molecular analyses of four patients in two families from Pakistan and Finland.

**Methods:**

Clinical analyses including radiography were completed and blood samples were collected. The extracted DNA was subjected to whole-exome analysis or target gene sequencing. Segregation analyses were performed in the nuclear pedigree. Pathogenicity prediction scores for the selected variants and conservation analyses of affected amino acids were observed.

**Results:**

The phenotype in the four affected individuals was consistent with multicentric osteolysis or MONA, as the patients had multiple affected joints, osteolysis of hands and feet, immobility of knee joint and progressive bone loss. Long-term follow up of the patients revealed the progression of the disease. We found a novel *MMP2* c.1336 + 2T > G homozygous splice donor variant segregating with the phenotype in the Pakistani family while a *MMP2* missense variant c.1188 C > A, p.(Ser396Arg) was homozygous in both Finnish patients. *In-silico* analysis predicted that the splicing variant may eventually introduce a premature stop codon in *MMP2.* Molecular modeling for the p.(Ser396Arg) variant suggested that the change may disturb MMP2 collagen-binding region.

**Conclusion:**

Our findings expand the genetic spectrum of Multicentric osteolysis nodulosis and arthropathy. We also suggest that the age of onset of this disorder may vary from childhood up to late adolescence and that a significant degree of intrafamilial variability may be present.

**Supplementary Information:**

The online version contains supplementary material available at 10.1186/s12891-023-06856-2.

## Background

Multicentric osteolysis, nodulosis and arthropathy (MONA, MIM #259,600) is a rare autosomal recessive skeletal disorder that is characterized by osteolysis of carpal and tarsal bones, formation of nodules under the skin of palms and soles and progressive osteoarthropathy (joint contractures, pain, swelling, and stiffness) [1, 2]. Other features may include coarse facies, pigmented skin and corneal opacities. Short stature occurs in most of the individuals, but the manifestation of short stature may occur due to progressive osteolysis of joints [3, 4]. MONA is caused by a deficiency of matrix metalloproteinase 2, (MMP2). Torg-Winchester syndrome is also included in the spectrum of MONA but nodules under the skin are absent. Another related disorder, Winchester syndrome (MIM# 277,950) is caused by variants of *MMP14* but some of the patients have variants in *MMP2* [5, 6]. MONA is a rare genetic disease with only 52 patients and 23 variants reported in the literature for the osteolysis syndrome [1–4, 7–19]. No epidemiological statistics of MONA have been reported.

MMP2, a 72 kDa protein encoded by *MMP2* is also known as gelatinase A and type IV collagenase. The zinc-dependent activities of the enzyme are capable of cleaving components of the extracellular matrix, cell surface receptors, growth factors and molecules involved in inflammation and signal transduction [20]. Specifically, MMP2 acts to degrade collagen and gelatin which is necessary for collagen turnover [21]. MMP2 is involved in normal tissue homeostasis, craniofacial development, bone cell growth and proliferation. This protein has a crucial rule for inflammatory bone and joint lesions as all MMPs contribute to inflammatory processes by regulating physical barriers, modulating inflammatory mediators such as chemokines and cytokines and establishing chemokine gradients in inflamed tissues. The MMP2 hemopexin domain binds to monocyte chemoattractant protein-3 (MCP-3) and causes it to cleave into a form that acts as a receptor antagonist rather than an agonist. The inflammatory phenotypes of *Mmp2* null mice also showed increased immune mediated arthritis along with other phenotypes [22].

One of the main functions of matrix metalloproteinase 2 is to cleave type IV collagen. Type IV collagen is a major structural component of the basement membranes, which are thin, sheet-like structures that separate and support cells as part of the extracellular matrix [18]. The extracellular matrix is highly organized by tissue remodeling in a fine tuned manner by protein formation and degradation. The known *MMP2* variants eliminate the function of the matrix metalloproteinase 2 enzyme, preventing the normal cleavage of type IV collagen. MMP2 is expressed as a latent proenzyme. It contains multiple domains like propeptide (residues 1–80), a catalytic domain (residues 81–192 and 368–436), three fibronectin type 2-like (FNII) domains (residues 199–247, 257–305, 315–363) and a hemopexin (PEX) domain (residues 442–631). There is a coordination of Cys73 of propeptide region to the catalytic zinc ion and if this links breaks, then the MMP2 is in its activated form. The activation is due to a proteolytic cleavage that triggers a conformational change in the cysteine switch of the propeptide. The cysteine switch is actually bound to the catalytic cleft by several hydrogen bonds and forms a loop structure. The cleavage occurs between these loops that renders MMP2 in its activated form [23, 24].

All previously reported *MMP2* variants, except for one, are frameshift, small deletions, insertions and missense. Here we report a novel canonical splice site variant in a consanguineous Pakistani family and a previously reported missense variant in a Finnish family diagnosed with MONA.

## Materials and methods

### Ethical approval

This study was approved by Institutional Review Board of School of Biological Sciences, University of the Punjab Lahore, Pakistan, and the Research Ethics Committee of the Hospital District of Helsinki and Uusimaa, Helsinki, Finland. Written informed consents were obtained from all participants.

### Subjects

**Fig. 1:**
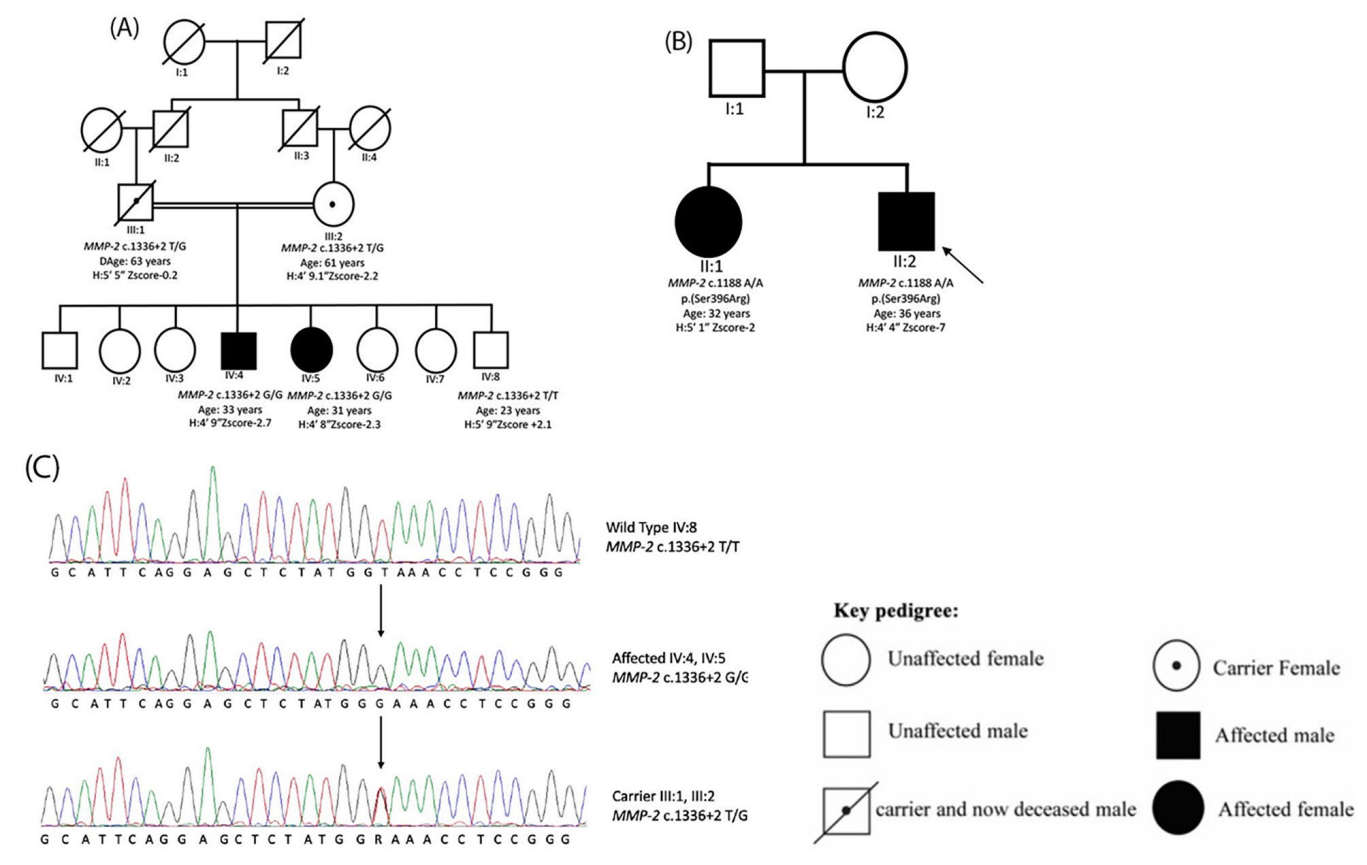
Pedigree and sequence traces of Family 1 and Family 2. **(A)** Pedigree of Family 1 (TID-08). Genotypes for *MMP2* variant c.1336 + 2T > G, ages and height Z scores are shown below the symbols of the participants. Filled circles and squares indicate affected female and male individuals respectively. Double line shows cousin marriage. Individual III:1, the father of the patients, died from a cardiac arrest at the age of 63 years, after the study was initiated. Paternity and maternity analyses were not performed for the patients. **(B)** Pedigree of family 2. Genotypes for c.1188 C > A, p.(Ser396Arg) variant of the two patients their ages and height Z scores are shown below each symbol. The parents were unrelated and their samples were not available. **(C)** Partial electropherograms of DNA sequence of *MMP2* c.1336 + 2T > G for wild type, affected and heterozygous individuals are shown. Arrows indicate the point of variation

Family 1 (TID-08) (Fig. 1A) was recruited from the Punjab province of Pakistan. Parents were first cousins. The photographs of hands, feet, arms, legs and faces of both patients were obtained for phenotypic characterization. Heights and head circumference of the participants were recorded. Family history was gathered from multiple individuals to confirm the consanguinity. Age of disease onset was ascertained by questioning. Radiographs were obtained from patient IV:4. Blood samples were collected from the available members of the family. DNA was extracted using a standard protocol by sucrose lysis and salting out. RNA was also extracted from blood samples using TriZol Reagent (Invitrogen™, Carlsbad CA, USA).

Family 2, with two affected siblings (Fig. 1B), was recruited from Helsinki University Hospital, Helsinki, Finland. The parents were of Finnish descent with no known consanguinity. Clinical and family histories were obtained. Blood samples were drawn from the affected siblings and DNA was extracted using standard procedures.

### Molecular analysis

In Family 1, exome sequencing was performed (3 billion Inc., Seoul, South Korea) on the DNA samples of both patients. Exonic regions of human genes were captured by using xGen Exome Research panel v2 (Integrated DNA Technologies, Coralville, lowa, USA). The captured regions were sequenced with NovaSeq 6000 (Illumina, San Diego, CA, USA). Alignment was completed using GRCh37/hg19 human reference genome. The vcf data was analyzed with Franklin software (https://franklin.genoox.com/clinical-db/home) as well as by 3 billion automated filters. First, all heterozygous and homozygous variants shared by both patients were obtained. Next, heterozygous or homozygous, autosomal recessive, missense, splicing, frameshift, as well as synonymous variants with an allele frequency of less than 0.01 in public databases including gnomAD (https://gnomad.broadinstitute.org/) and 1000 genomes (https://www.ncbi.nlm.nih.gov/snp/?term=1000genomes) were prioritized. Prioritization was also performed based on the pathogenicity scores. Pathogenicity scores of the variants from REVEL, SIFT, PolyPhen2, MutationTaster, DANN and splice site prediction scores were assessed from SpliceAI, dbscSNV Ada and dbscSNV RF available through Franklin software. The shortlisted pathogenic candidate variant was checked using Sanger sequencing of DNA from all participants (Fig. 1C). The primers (Supplementary Table 1) for candidate gene sequencing and cDNA synthesis were designed using primer 3.0 software (https://bioinfo.ut.ee/primer3-0.4.0). cDNA libraries were prepared from RNA extracted with TriZol from human blood samples using both random hexamer and oligo dT primers according to a standard protocol (Thermo Fisher Scientific RevertAid first strand cDNA synthesis kit). Primers for *GAPDH* and *MRM2* amplification (Supplementary Table 1) were used as positive controls.

In Family 2, Sanger sequencing of the *MMP2* gene was performed using the DNA sample from the index patient. *MMP2* variant testing was subsequently carried out on the DNA sample from the affected sister. The sequencing was completed at Blueprints Genetics, Helsinki, Finland. DNA samples from the parents were not available. The allele frequency of the identified missense variant was observed in gnomAD (https://gnomad.broadinstitute.org/) and SISu project (https://sisuproject.fi/).

### *In Silico* Analysis and conservation

The splice donor *MMP2* variant c.1336 + 2T > G in Family 1 was analyzed by prediction tool.

Human Splice Finder (HSF)( https://hsf.genomnis.com) and (www.fruitfly.org/seq_tools/splice.html) [25]. Conservation of amino acid affected by the missense *MMP2* variant c.1188 C > A, p.(Ser396Arg) was determined by Clustal Omega (https://www.ebi.ac.uk/Tools/msa/clustalo/) using vertebrate orthologue protein sequences obtained from UniProt (https://www.uniprot.org/uniprotkb?query=mmp2) for human, mouse, rat, chicken, lizard, frog and fish. The HOPE tool (https://www3.cmbi.umcn.nl/hope) was used to predict the structural effect of the detected amino acid change on the protein conformation using the MMP2 sequence P08253.

## Results

### Clinical findings

**Fig. 2:**
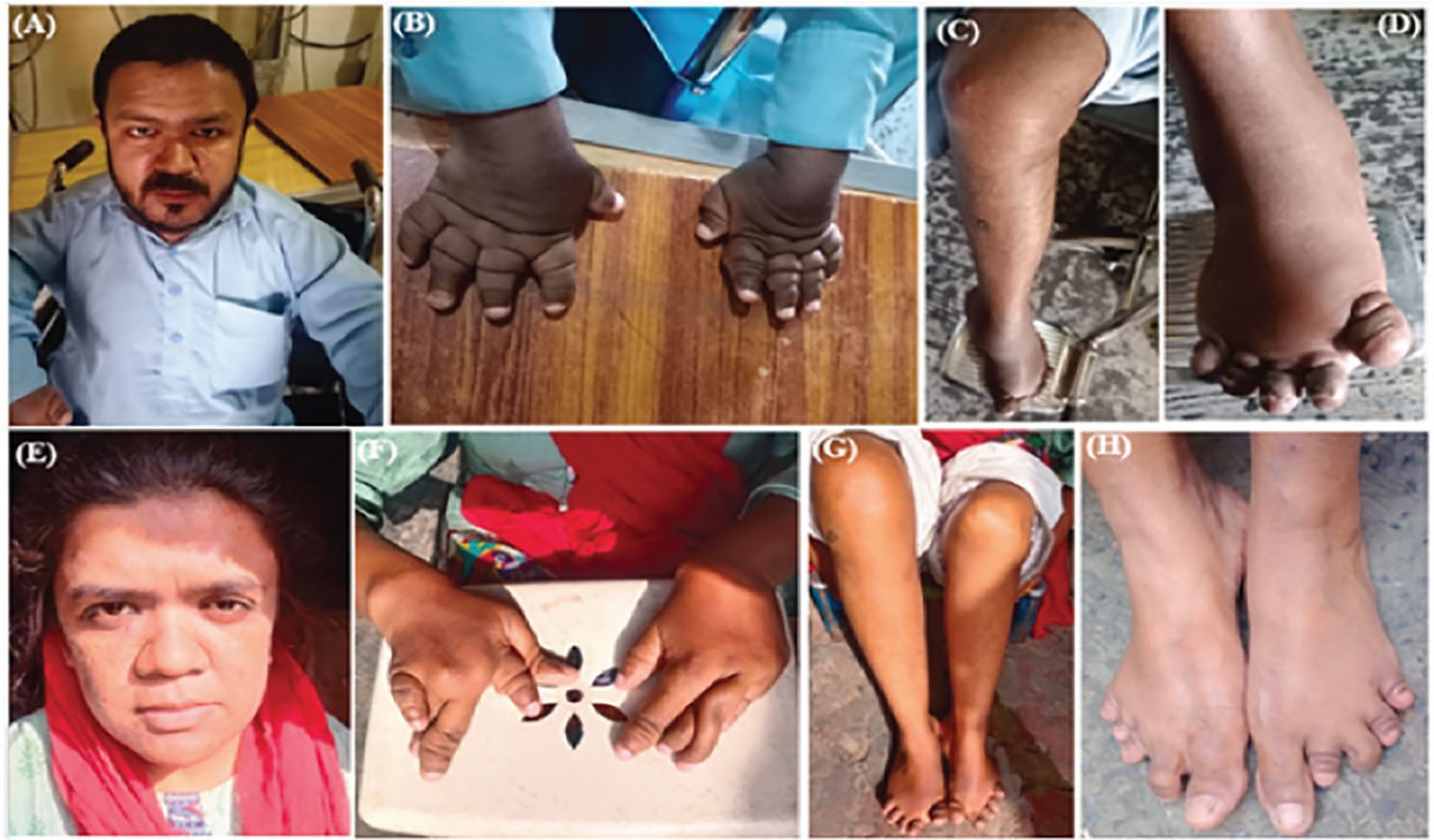
Phenotypes of affected individuals of Family 1. **(A)** Facial features of patient IV:4. Bulbous nose with flattened nasal bridge, broad philtrum, and coarse face. **(B)** Patient IV:4 hands deformity. **(C)** Patient IV:4 swollen knee joint with immobility **(D)** Patient IV:4 swollen feet with small constricted fingers. **(E)** Patient IV:5’s facial features showing coarse face, flat nasal bridge with bulbous nose and hirsutism. **(F)** Patient IV:5 curved and deformed fingers of hands. **(G)** Swollen knee joints **(H)** Constricted feet fingers

**Fig. 3:**
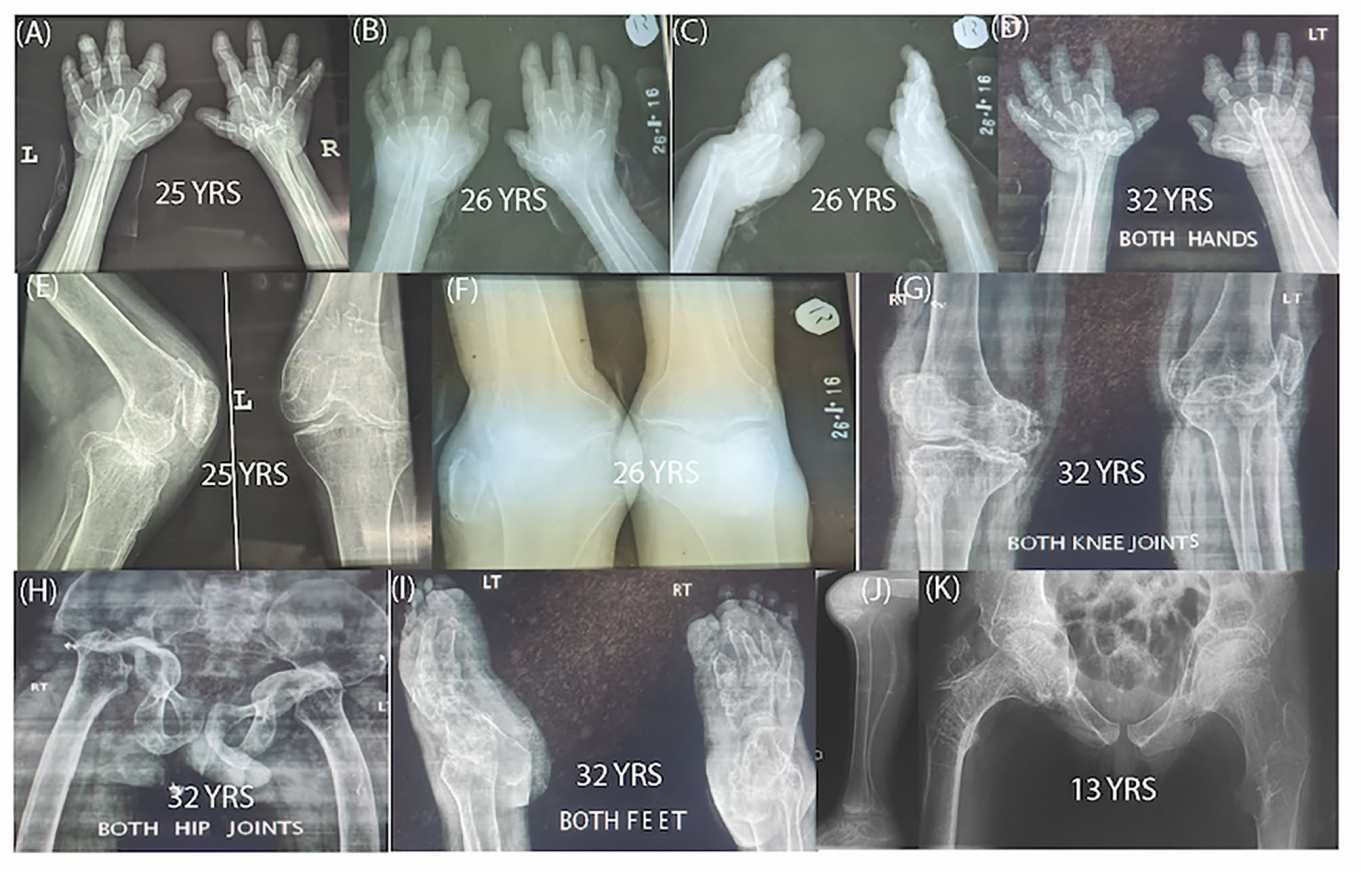
Radiographs of Family 1 and Family 2. Radiographs of patient IV:4 at age 25 years showing osteopenia, deformed wrist, distal radioulnar joint cortical thinning and bullet shaped metacarpals **(B)** Radiograph at age 26 shows increased osteolysis, poor visualization of carpal bones, cortical thinning of metacarpal bones and medullary expansion due to advanced osteopenia **(C)** Radiograph of the same patient at the age of 26 years, vertical view, showing protrusion of radius and ulna of the left hand **(D)** Radiograph of the same patient at age 32 showing complete osteolysis of carpal bones and abnormal shape of the metacarpal bones. Pencil-in cup deformity of most of proximal and distal interphalyngeal joints due to osteolysis is also visible **(E)** Radiograph of the knee joint at age 25 years showing marked narrowing of medial joint space of knee **(F)** At the age of 26 years, the knee showed more narrowing of space and progressive osteolysis **(G)** At the age of 32 years, the left knee joint shows destruction of the surfaces. The joint is locked; unable to move, with early osteoarthritic changes due to continuing osteolysis **(H)** Hip joint at the age of 32 years showing periarticular and generalized osteopenia osteonecrosis. There is complete destruction of femoral heads advanced protrusion acetabula bilaterally leading to deformed pelvic shape. Shepherd crock deformity of left femur is visible **(I)** Deformity and coalition of tarsal bones. **(J, K)** Radiographs of the Finnish male patient at age 13 years. Images show severe generalized demineralization and bowing of the very thin fibula. The knee and ankle joints show osteolytic changes and destruction of the joint surfaces. Proximal femurs are similarly osteopenic, femoral necks are thin and acetabuli and femoral epiphyses are irregular

Two individuals in Family 1 (TID-08) were affected with multicentric osteolysis nodulosis and arthropathy. The individuals were born to unaffected parents and were reported to be asymptomatic until the age of 16 years. The affected individuals IV:4 (male, age 33 years) and IV:5 (female, age 31 years) have heights of 150 cm (-2.7 SD) and 146 cm (-2.3 SD) respectively. Facial features include coarse faces, proptosis, frontal bossing, hypertelorism, and a bulbous nose with a flattened nasal bridge (Fig. 2A-H). The disorder was progressive as the symptoms started at 16 years of age with deformity of the hands and feet visible on radiographs and osteolysis of carpal and tarsal bones. The deformity of hands and feet gradually progressed with age as indicated by the sequential radiographs of the male patient at the ages of 25, 26 and 32 years (Fig. 3A-D). The problems led to knee joint immobility. The patients became wheel chair bound (Fig. 2) due to early osteoarthritic changes of continuing osteolysis (Fig. 3E-G). No intellectual disability was present in the patients and they had attended school until 16 years of age. At 17 years of age the male patient was diagnosed with pariarticular juvenile idiopathic arthritis. He was started on anti-rheumatic drugs leflunamide and plaquenil with no obvious results for five years. After 5 years he was also given methotrexate and nivaquin. None of the treatment stopped the progressive osteolysis, but rather the pain was relieved with painkillers.

The affected female in Family 1, now aged 31 years was also diagnosed with the same disease as her brother at the age of 17 years. She was treated with similar medications without any improvement in her disorder. Unlike her brother, she exhibited gingival hypertrophy. She had a normal menstrual cycle and did not suffer from polycystic ovary syndrome.

In Family 2, the index patient is a 36-year-old male born to healthy parents. He had a normal perinatal period and his developmental milestones were normal. He presented with swelling, pain and flexion contracture in the left wrist at the age of 2 years. Osteoporosis was revealed by X-rays following a radius fracture at 2 years of age. Minor facial dysmorphism was observed, such as a flat nasal bridge, thick eyebrows with partial synophrys and a broad philtrum. At 3 years of age, he suffered from pain and swelling of right foot. He had progressive deformity of small and larger joints and developed osteolysis of feet and hands, scoliosis and hip deformity. At 17 years of age, his bone mineral density (BMD) Z-score for the lumbar spine was − 6.0 and he was started on bisphosphonate treatment, which he continued until the age of 34.

The Family 2 proband has had several treatment attempts of variable duration with bisphosphonates, first because of osteopenia and more recently to slow down osteolysis. He first received zoledronic acid from ages 17 to 21 years (4 mg/year). After a two-year treatment pause, oral ibandronic acid (150 mg/month) was started but compliance was uncertain. At the age of 27 years he commenced oral alendronate (70 mg/week). BMD Z-score for lumbar spine at 36 years of age was − 1.5. Several orthopedic surgeries of hip, spine, knee and ankles were performed during adolescence and adulthood. Intelligence was normal. Ophthalmological examination revealed signs of cataract. Presently, he has a short stature with a height of 135 cm (-7 SDS) and uses a walker and a wheelchair.

The affected sister in Family 2 is 32 years of age. She had normal developmental milestones and her growth was normal. Flexion contractures in the fingers were noticed at the age of 4 years. Joint contractures developed over time to include small and larger joints. She was diagnosed with polyarthritis at 12 years of age and has received anti-rheumatic drugs (hydroxychloroquine, etanercept) without response. Occasional local intra-articular cortisone injections (knees, shoulders, wrists) transiently alleviated the pain. Orthopedic surgeries of feet and hands were performed during adolescence. She had plantar and palmar subcutaneous nodules. She received bisphosphonate treatment from the age of 14 years without major improvement. Intelligence was normal. Her height is 157 cm (− 2.0 SDS) and she uses a wheelchair. Ophthalmological examination revealed corneal opacities. She also had polycystic ovary syndrome.

### Radiographic findings

X-Ray images of the affected individual IV:4 (age 33) of Family 1 showed several skeletal anomalies of hands, feet, hips and knees. Radiographs taken at the ages of 25, 26 and 32 years respectively, showed flexion contracture, diffused osteopenia, cortical thinning, osteolysis and multiple fractures with bullet shaped metacarpal bones of both hands. Carpal bones were not separately visualized; irregular deformed phalanges with ‘pencil-in cup’ deformity and pseudoarthrosis were observed. Osteolysis increased with time which was obvious from radiographs taken at different ages. Poor visualization of carpal bones also suggested osteolysis of these bones. Metacarpal bones depicted cortical thinning and medullary expansion as well due to advanced osteopenia. Wrist and distal radio ulnar joint showed fusion and deformity (Fig. 3A-D). Feet also exhibited marked osteopenia with cortical thinning, deformity and coalition of tarsal bones and anterior beaking of metatarsal bones. The phalanges were tubular and small (Fig. 3I). Knee joint showed periarticular osteopenia, osteophytosis, marked narrowing of medial joint space and shallow intercondylar notch. Knee joint had early osteoarthritic changes due to continuing osteolysis (Fig. 3E-G). Hip joint had marked periarticular and generalized osteopenia with complete osteolysis/osteonecrosis of femoral heads, advanced acetabuli and deformed pelvis. Lateral bowing or shepherd crock deformity of bilateral femoral shafts and pubic diastasis were also observed (Fig. 3H).

**Fig. 4:**
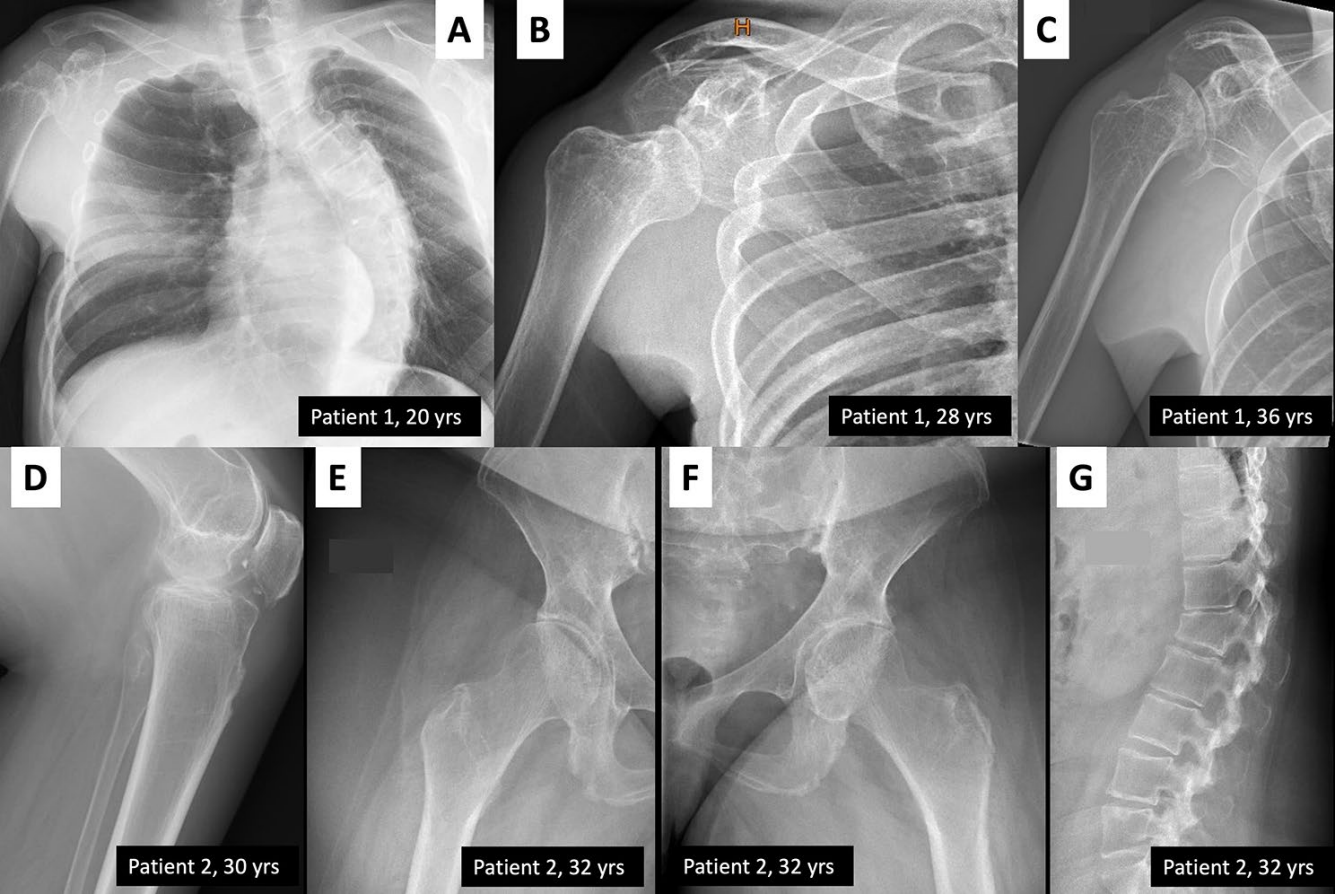
Progression of skeletal changes during adulthood in patients of Family 2. **(A, B, C)** Radiographs of Patient 1 at ages 20, 28 and 36 years, showing severe scoliosis and progressive deformity and osteolysis of the proximal humerus. **(D, E, F, G)** Radiographs of Patient 2 at ages 30 and 32 years show generalized osteopenia, osteolysis and osteoarthritis in the knee and hip joints, and a vertebral compression fracture in the spine

Radiographs of the patients in Family 2 showed significant generalized osteopenia and osteolysis especially in the male patient, who had severe skeletal manifestations already at adolescence (Fig. 3J-K). Manifestations progressed with age (Fig. 4A-C). The female patient was less severely affected, in line with a less severe clinical course (Fig. 4D), but at 32 years she had signs of osteolysis and osteoarthritis in the knee and hip joints, as well as a vertebral compression fracture (Fig. 4E-G).

### Molecular analysis findings

There were 1214 heterozygous and 445 homozygous variants located in the coding and the non-coding regions which were present in the exome data of both patients of Family 1 (data not shown). Filtering the data revealed 12 homozygous variants (Supplementary Table 2) and 132 heterozygous variants (Supplementary Table 3). None of the latter affected genes, variants of which are known to be correlated with the patients’ phenotype. Out of the 12 homozygous variants, all but three variants were discarded since they were present in the public databases as homozygous or in a frequency of > 1% in samples of 400 unrelated ethnically matched controls from Pakistan (Supplementary Table 2). Of the remaining variants, one was a missense variant in (NM_001185024.1) *ZDHHC8* c.1575 C > A, p.(Phe525Leu) while another was a missense variant in (NM_004530.6) *MMP2* c.344G > T p.(Arg115Leu). These variants were not considered further since they were both predicted to be non-damaging to the protein by multiple software and affected amino acids which are not conserved in evolution (Supplementary Table 2). Only one possible candidate variant in (NM_004530.6) *MMP2* c.1336 + 2T > G remained which was classified as “pathogenic” according to the ACMG guidelines meeting criteria PVS1, PM2, PM3, PP1, PP3 and PP4. Sanger sequencing confirmed the segregation of this variant with the phenotype as both patients were homozygous for the variant while the parents were heterozygous and the unaffected brother carried only the wild-type allele (Fig. 1C).

The *MMP2* c.1336 + 2T > G variant was very rare with a frequency of 0.000025 and 0.000016 in ExAC and gnomAD database respectively with no homozygotes in any database. Of note, the only four heterozygous carriers were South Asians (https://gnomad.broadinstitute.org/variant/16-55525870-T-G?dataset=gnomad_r2_1). This suggests that this mutation could have arisen in this population in a common ancestor. The variant was absent from DNA samples of 400 ethnically matched controls (800 chromosomes). Using the cDNA primers we tried to check effect of the donor splice variant on *MMP2* splicing, but there was no amplification of cDNA from mRNA even on that obtained from blood samples of healthy controls. This may suggest that either this gene is not expressed in blood in appreciable quantitites at mRNA level or both primer pairs were not optimal. Succesful amplification of two positive controls (*GAPDH* and *MRM2*) verified the integrity of the prepared cDNA libraries.

**Fig. 5:**
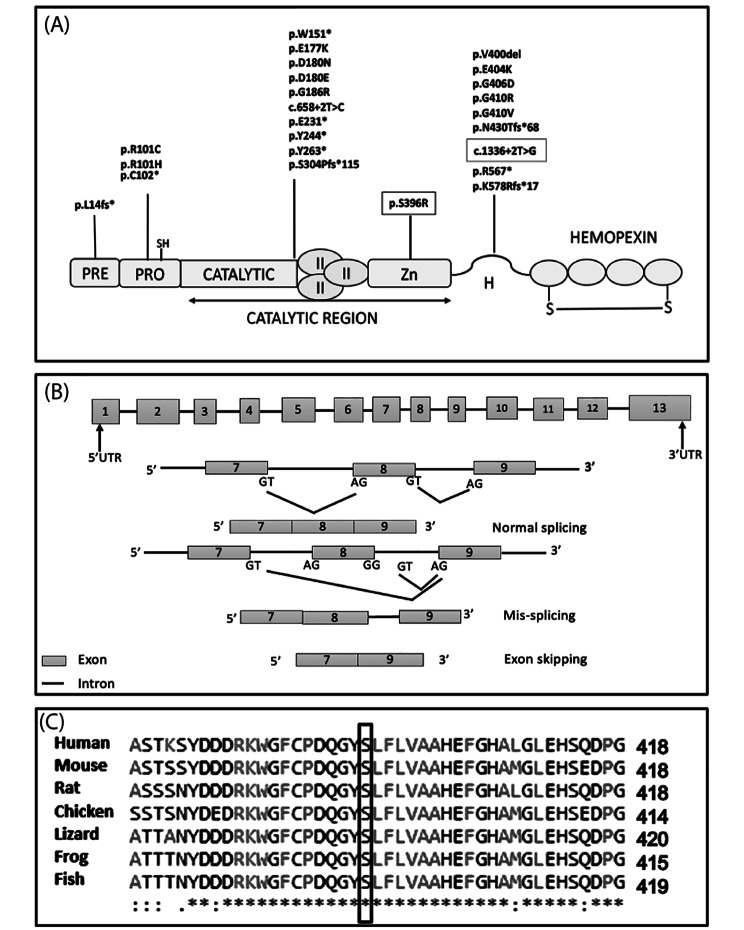
MMP2 domains, predicted splicing effects and clustal alignment. **(A)** Domain structure of MMP2 showing all disease-causing variants reported so far. Pre: signal peptid, Pro: propeptide with a free zinc ligating thiol group, II: collagen binding fibronectin type II inserts, Zn: zinc binding site, H: hinge region, emopexin/vitronectin-like domain contains four repeats with the first and last linked by disulfide bond. Variants highlighted in rectangles were reported in this paper. **(B)** Predicted Mis-splicing of *MMP2* exon 8 due to splice site variant 1336 + 2T > G in family 1.*MMP2* showing all 13 exons. Box represent the exons and lines show the introns. Normal splicing GT is recognized by AT and the intron is removed while 1336 + 2T > G variant will result in either a part of intron 8 to be added to the transcript due to mis-splicing or the whole exon 8 to be skipped. **(C)** Multiple sequence alignment of conserved amino acid Serine 396 in family 2. Multiple sequence alignment of amino acids from human, mouse, rat, chicken, lizard, frog and fish using the protein sequence obtained from UniProt and aligned using Clustal Omega showing the conserved amino acids. Serine 396 is highlighted and is completely conserved in all species

Various pathogenicity prediction programs had suggested that the c.1336 + 2T > G variant was strongly deleterious with Splice AI, dbscSNV Ada and dbscSNV RF scores ranging from 0.94 to 1 (Supplementary Table 2). This variant affects splicing of exon 8 which encodes the hinge region of MMP2 (Fig. 5A). *In silico* prediction tools indicated that this variant will cause mis-splicing of exon 8 by using another alternative donor splice site within intron 8 (Fig. 5B), with high prediction scores of 0.99 and 80.7 (Splice Site Prediction by Neural Network and HSF respectively). If indeed this is the case, the addition of the few extra nucleotides will introduce a premature stop codon in *MMP2* (Fig. 5B). Nonsense mediated mRNA decay process may occur, resulting in loss of *MMP2* transcript [26].

**Fig. 6:**
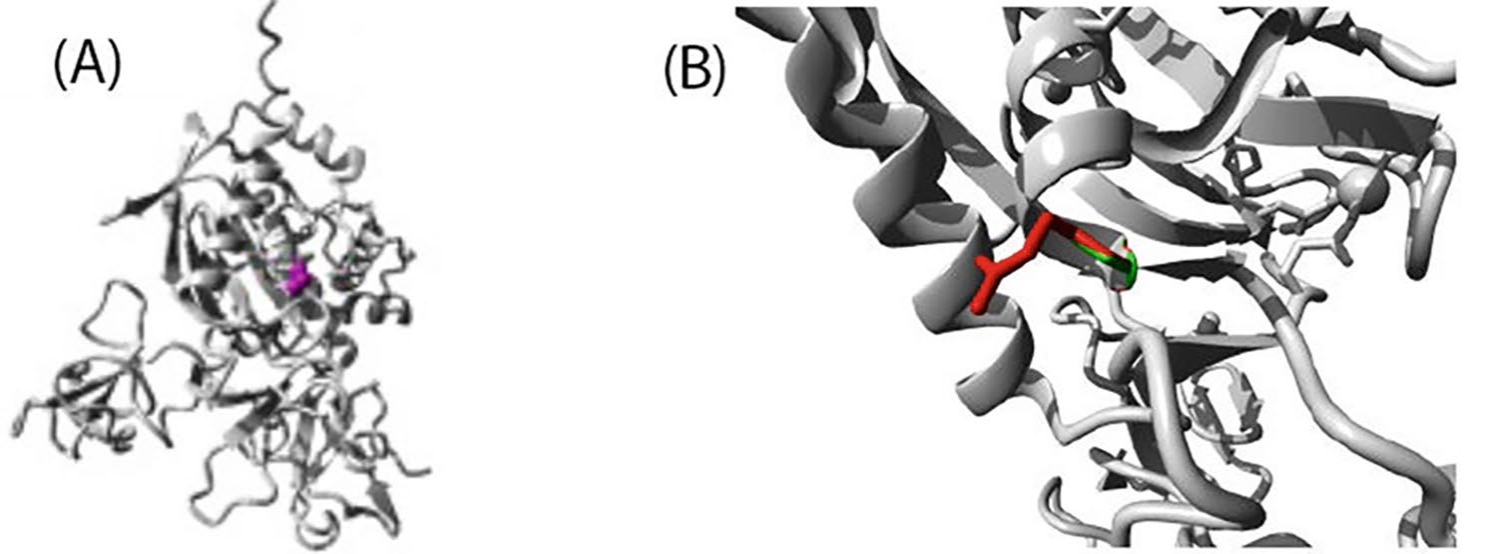
MMP2 protein and effect of variant on structure. **(A)** Overview of the protein in ribbon-presentation. The protein is coloured grey, the side chain of the mutated residue is coloured magenta and shown as small balls. **(B)** Close-up of the mutation. The protein is coloured grey, the side chains of both the wild-type and the mutant residue are shown and coloured green and red respectively

In Family 2, sequencing of the *MMP2* gene in the index patient revealed a homozygous missense variant in *MMP2* (NM_004530.6): c.1188 C > A, p.(Ser396Arg). The same homozygous variant was found in the affected sister. It was classified as a “variant of unknown significance” by ACMG guidelines but was predicted as damaging/pathogenic by SIFT, M-CAP, MutationTaster2, PROVEAN and PrimateAI. CADD score was 25 and PolyPhen2 predicted the variant as probably damaging. The identified variant was present with an overall gnomAD allele frequency of 0.0001591. However, the Finnish specific allele frequency is higher; 0.0017 (43/25,122, all heterozygotes) and 0.00176 (37/10,453, all heterozygotes), in the gnomAD and SISu project, respectively which suggests enrichment in this population. This variant affects an amino acid Ser 396 which is conserved in vertebrate species (Fig. 5C). Analysis of the mutant protein by HOPE showed that the mutant residue is bigger, more hydrophilic and introduces a positive charge compared to the smaller, neutral charged wild-type residue (Fig. 6). The analysis also indicated that the variant affects the collagen binding domain of MMP2 which may thus disrupt function.

## Discussion

We describe four individuals from two different families exhibiting severe form of multicentric osteolysis, nodulosis, and arthropathy (MONA). Biallelic variants in *MMP2* were identified in all affected members of these families as the cause of the disease.

*MMP2* (NM_004530.6) consists of 13 exons and is located on chromosome 16q12-q21 [15]. The gene encodes a 660 amino acid protein that consists of multiple functional domains, including signal peptide, propeptide domain, catalytic domain, hemopexin domain, three contiguous fibronectin like domains and a highly conserved cysteine residue which is essential to maintain the MMP2 in an inactive state (Fig. 5A) [23]. The *MMP2* variants reported so far in the literature disrupt the activity of MMP2 by loss of function (Fig. 5A). *MMP2* variants were previously reported in NAO and Torg-Winchester syndrome. All of these names overlap with the phenotypes of the patients having more than one body parts affected and presence of nodules under the skin or presenting all phenotypes together [2]. Multicentric Osteolysis, Nodulosis, and Arthropathy (MONA MIM #259,600) is the best term to describe the phenotypes together due to *MMP2* variants as a cause of disease in our patients (Table 1).

**Table 1:** Clinical findings of patients in Families 1 and 2

Clinical findings	Family 1		Family 2	
	Male patient IV:4	Female patient IV:5	Male patient II:2	Female patient II:1
Osteolysis, and location	Yes, generalized	Yes, generalized	Yes, generalized	Yes, generalized
Osteoporosis/fractures	Yes	Yes	Yes	Yes
Progressive arthropathy	Yes	Yes	Yes	Yes
Joint contractures	Yes	Yes	Yes	Yes
Pain	Yes	Yes	Yes	Yes
Swelling	Yes	Yes	Yes	Yes
Stiffness	Yes	Yes	Yes	Yes
Scoliosis/ kyphosis	No	No	Yes	Yes
Bowing deformities	Yes	Yes	Yes	Yes
Ophthalmologic findings	No	No	Cataract	Corneal opacities
Gingival hypertrophy	No	Yes	No	No
Dental abnormalities	No	No	No	No
Cardiac defects	No	No	No	No
Hirsutism	No	Yes	No	No
Subcutaneous nodules and location	No	Yes, palmar and plantar	No	Yes, palmar and plantar
Pigmented skin lesions	Yes	Yes	No	No

The splice donor variant c.1336 + 2T > G reported here is located within intron 8 of the 13 exon long gene. Due to the predicted cryptic mis-splicing and addition of a part of intron into the final mRNA, the premature stop codon will likely mark the RNA for nonsense mediated mRNA decay (NMD) [26]. This prediction is similar to the previously reported *MMP2* variant c.732 C > A, p.(Tyr244Ter) that introduces a premature stop codon and triggers nonsense-mediated mRNA decay. Therefore, no MMP2 activity was found in serum and fibroblast of patients homozygous for this variant [15]. If the variant causes exon skipping or if the mis-splicing occurs and NMD does not take place, a smaller protein such as described earlier for the *MMP2* variant c.658 + 2T > C, that causes skipping of exon 4 [11], may be produced. This disturbs the catalytic domain of MMP2 which is essential for its activation [23]. The segregation of the splicing variant c.1336 + 2T > G in patients with the disorder, and its absence in ethnically matched controls suggests that the variant is likely pathogenic. We were unable to verify the effect of the splicing variant due to a lack of patient fibroblast samples and an inability to amplify *MMP2* cDNA from blood samples.

We also showed the age of onset of this osteolysis syndrome beyond the age already reported between birth to 11 years [1]. In both our patients in Family 1, the symptoms first appeared at age 16 years and progressed until the patients were bound to wheel chair. Since the splice donor *MMP2* variant detected in Family 1 is a likely loss of function mutation, the later onset of the disease in these patients in comparison to the other described affected individuals suggests that a different variant may act as a modifier in conferring a delayed onset of the disease. In this regard, prior to filtration, there are 444 homozygous and 1214 heterozygous variants in the exome data which are identical in both patients. Any of these could be a potential candidate as a modifier variant.

The *MMP2* missense variant Ser396Arg has previously been detected as a homozygous variant in two siblings in a Finnish family [13] and as a compound heterozygous variant in one European patient with MONA phenotypes [8]. Kröger et al. analyzed the level of MMP2 in serum in their index patient, homozygous for the Ser396Arg variant, by gelatin zymography and found that MMP2 was non-measurable. They also examined bone histomorphometry, which suggested increased trabecular bone remodeling and turnover. The patients in their study were of younger age and had a shorter follow-up as compared to the patients in Family 2 in our study. The age of onset of the disease in their index patient and our patients were similar. Our index patient had severe short stature with height − 7 SDS, compared with their patients’ heights − 0.1 to -1.1 SDS and − 1.5 SDS. There were also some other phenotypic differences such as the pigmented skin lesions, skin thickening, gingival hypertrophy and severe corneal opacities with visual impairment described in their index patient [13]. These findings show that there may be high degree of phenotypic variability in patients carrying the same variant.

The missense variant Ser396Arg is more common in Finland (allele frequency 0.17%) compared to other populations (0.016%). This allele frequency is well in line with, or even lower than the frequency of pathogenic variants of other autosomal recessive diseases of the Finnish heritage [27]. Taken together, these findings suggest that the p.Ser396Arg variant maybe a founder mutation in Finland giving rise to MONA. However, in the absence of experimental data to prove its pathogenicity, the relatively high frequency of the p.Ser396Arg variant in the Finnish population and the population structure favoring remote consanguinity [28] suggest that this mutation could be present as homozygous in the patients by chance.

## Conclusion

In conclusion, our patients fall in the phenotypic spectrum of Multicentric Osteolysis Nodulosis and Arthropathy (MONA) syndrome which is allelic to Torg-Winchester and NAO syndromes. We expand the mutational spectrum of *MMP2.* We also suggest that the age of onset of this disorder may vary from childhood up to late adolescence and that a significant degree of intrafamilial variability may be present.

### Electronic supplementary material

Below is the link to the electronic supplementary material.

Supplementary Material 1

## Data Availability

All data generated or analysed during this study are either included in this published article and its supplementary information files or are available from the corresponding author on reasonable request. The novel splicing variant is deposited in LOVD as record # 0000921592.
