# Supplementary material for: Clinical, radiographic and molecular characterization of two unrelated families with multicentric osteolysis, nodulosis, and arthropathy
Source: BMC Musculoskelet Disord. 2023 Sep 14;24:735. doi: 10.1186/s12891-023-06856-2 (PMC10503101; doi:10.1186/s12891-023-06856-2)
Supplement: Supplementary file 1 — Supplementary Material 1 [file 12891_2023_6856_MOESM1_ESM.docx]

**Supplementary material**

**Supplementary Table 1: Genomic DNA and cDNA primers**

| **Primer name** | **Region** | **Sequence** |
| --- | --- | --- |
| Forward | Genomic DNA | 5ʹ-CCAGGGTTCTCAGCCTTACTGTGGGGCTGT- 3ʹ |
| Reverse | Genomic DNA | 5ʹ-CTGCTCTGCCTTCCCAAGACCAGGTCAACA-3ʹ |
| Outer forward | cDNA | 5ʹ-CTGCCCTGAGACCGCCATGTC-3ʹ |
| Outer reverse | cDNA | 5ʹ-TGAAGAAGTAGCTGTGACCGCCGC-3ʹ |
| Inner forward | cDNA | 5ʹ-CCTGACCAAGGGTACAGCCTGTTCCT-3ʹ |
| Inner reverse | cDNA | 5ʹ-GCTGAGTAGATCCAGTATTCATTCCCTGCA-3ʹ |
| *Control forward | cDNA | 5ʹ-AGGCGCGGGGAACACCAAT-3ʹ |
| *Control reverse | cDNA | 5ʹ-TTCCTGCTGGCTTCAGGTTTGATG-3ʹ |

The first six primers are specific to *MMP2*. **MRM2* specific primers were used as control for amplification from the cDNA libraries. Proprietary primers amplifying *GAPDH* cDNA were used as a second control.

**Supplementary Table 2: Homozygous variants after filtration from exome data analysis of Family 1**

| **Gene** | **Position (hg19)** | **Transcript, cDNA change and Effect** | **gnomAD AF** | **Prediction** | **Comments** |
| --- | --- | --- | --- | --- | --- |
| *CRIPAK* | chr4: 1388759 | NM_175918.3,  c.460C>T,  p.Arg154Trp | 0.0015581178 | Variant of uncertain significance by ACMG | Discarded, high internal frequency, 110 homozygous individuals in control population |
| *MMP2* | chr16: 55525870 | NM_004530.6,  c.1336+2T>G,  splice donor* | 0.00001607 | Predicted to be pathogenic by multiple software, Pathogenic by ACMG | Prioritized |
| *MMP2* | chr16: 55517011 | NM_004530.6,  c.344G>T,  p.Arg115Leu | 0.000223 | Predicted to be non-damaging to the protein by multiple software , Variant of uncertain significance by ACMG | Discarded, non-damaging  to protein, and affecting an amino acid not conserved in evolution (Monkeys have Asn while parrots have Gly in the corresponding position) |
| *MUC19* | chr12: 40884241 | NM_173600.2,  c.17501_17502insCATCAGCTGCTGTGACTGGGTCAGCTGGACTATCAGCTGGGGTGACAGGGACAACTGGAC,  p.Thr5834_Val5835insIle  SerCysCysAspTrpValSer  TrpThrIleSerTrpGlyAsp  ArgAspAsnTrpThr | 0 | Variant of uncertain significance by ACMG | Discarded, high internal frequency, 95 homozygous individuals in control population |
| *MYOM2* | Chr8: 2020377 | NM_003970.4,  c.794-47_794-8dupATGCTCTC  TGCCCTTCGGCCCTGA  AAGCCTCCATCGTTTC,  Splice region | N/A | Variant of uncertain significance by ACMG | Discarded, high internal frequency including greater than 100 unrelated homozygotes |
| *OR52N5* | chr11: 5799330 | NM_001001922.2,  c.535C>G,  p.Gln179Glu | 0 | Predicted to be likely benign, Likely benign by ACMG | Discarded, homozygous in 1 control and 1 internal control samples. Olfactory receptor gene |
| *PALD1* | chr10: 72288964 | NM_014431.3,  c.186-3delC,  splice region | 0.00149476 | Variant of Unknown Significance by ACMG | Discarded, homozygous in 5 controls and one internal control |
| *XKR3* | Chr22: 17264904 | NM_175878.4,  c.985T>C,  p.Leu329Leu | 0 | Variant of Unknown Significance by ACMG | Discarded, homozygous in 3 controls, high internal allele frequency with 42 homozygous individuals |
| *XKR3* | Chr22: 17265194 | NM_175878.4,  c.695C>T,  p.Pro232Leu | 0 | Likely benign by ACMG | Discarded, high internal frequency with 42 homozygous individuals |
| *ZDHHC8* | chr22: 20130728 | NM_001185024.1,  c.1575C>A,  p.Phe525Leu | 0.00007890 | Predicted to be benign by multiple software , Variant of Unknown Significance by ACMG | Discarded, non-damaging to protein and affecting an amino acid not conserved in evolution. The corresponding amino acids is Tyr or Ala in fish |
| *ZNF26* | chr12: 133587980 | NM_019591.3,  c.1515C>T,  p.Thr505Thr | 0 | Likely benign by ACMG as well as in ClinVar | Discarded, high internal frequency with more than 100 homozygous individuals |
| *ZNF84* | chr12: 133634450 | NM_001289972.1,  c.1149A>C,  p.Ala383Ala | 0 | Variant of Unknown Significance by ACMG | Discarded, high internal frequency with more than 200 homozygous individuals |

Genomic coordinates are provided according to GRCh37/hg19 genome assembly. *c.1336+2T>G Splice AI, dbscSNV Ada and dbscSNV RF prediction scores were 1, 1 and 0.94 respectively which are highly deleterious

**Supplementary Table 3:** Heterozygous variants after filtration of exome data from Family 1 patients

| **Gene** | **Chr** | **Position** | **Ref** | **Alt** | **Transcript** | **AA Change** | **Nucleotide** | **gnomAD AF** | **SIFT** | **PolyPhen2** | **GERP** |
| --- | --- | --- | --- | --- | --- | --- | --- | --- | --- | --- | --- |
| *A2ML1* | 12 | 9020917 | C | T | NM_144670.6 | p.Thr1342Ile | c.4025C>T | 0.000354 | U | B | 1.7 |
| *ABCA10* | 17 | 67190517 | T | C | NM_080282.3 | | c.1345+9A>G | 0.001155 |  |  |  |
| *ABCA6* | 17 | 67081218 | C | T | NM_080284.3 | p.Ala1379Thr | c.4135G>A | 0.002529 | U | U | 2.77 |
| *ACSL5* | 10 | 114170391 | C | G | NM_016234.3 | p.Thr321Arg | c.962C>G | N/A | D |  | 5.39 |
| *ACVR1B* | 12 | 52345540 | G | A | NM_004302.5 | p.Ala5Thr | c.13G>A | N/A | U |  | 2.58 |
| *ADAMTS15* | 11 | 130332104 | G | A | NM_139055.3 | p.Ala405Thr | c.1213G>A | 9.16E-05 | U | U | 5.59 |
| *ADGRF1* | 6 | 46979856 | G | A | NM_153840.4 | p.Arg335Trp | c.1003C>T | 0.000131 | U | U | 0.815 |
| *AGAP3* | 7 | 150840442 | G | A | NM_031946.7 | p.Arg763His | c.2288G>A | 6.86E-05 | U | U | 2.32 |
| *AGBL3* | 7 | 134674025 | G | A | NM_178563.4 | p.Val30Ile | c.88G>A | 1.95E-05 | U | U | 1.48 |
| *AKAP6* | 14 | 33015094 | G | A | NM_004274.5 | p.Arg412Lys | c.1235G>A | 0.000124 | B | B | 0.873 |
| *APH1B* | 15 | 63597890 | C | A | NM_031301.4 | p.Phe228Leu | c.684C>A | 0.000171 | U | U | 1.66 |
| *ARL14EPL* | 5 | 115394588 | A | G | NM_001195581.1 | p.Ile135Val | c.403A>G | 5.94E-05 |  | U | 4.8 |
| *ASB13* | 10 | 5683835 | C | T | NM_024701.4 | p.Glu203Lys | c.607G>A | 1.19E-05 | U | U | 2.97 |
| *BHLHE41* | 12 | 26277469 | T | C | NM_030762.3 | p.Lys37Glu | c.109A>G | N/A | U |  | 4.2 |
| *BMP7* | 20 | 55841113 | G | C | NM_001719.3 | p.Phe22Leu | c.66C>G | 0 | B |  | 2.71 |
| *BPTF* | 17 | 65941833 | G | A | NM_182641.4 | p.Gly2337Arg | c.7009G>A | 5.96E-05 | U | D | 4.99 |
| *BRIX1* | 5 | 34918515 | G | A | NM_018321.3 | p.Gly69Glu | c.206G>A | 8.05E-06 | U | D | 5.71 |
| *BRWD1* | 21 | 40570823 | A | G | NM_018963.4 | p.Met1840Thr | c.5519T>C | N/A | B |  | -1.88 |
| *C21orf91* | 21 | 19169012 | C | G | NM_001100420.2 | p.Arg184Pro | c.551G>C | N/A | B |  | -1.42 |
| *C9orf129* | 9 | 96080819 | T | C | NM_001098808.1 | p.His151Arg | c.452A>G | 0.000158 | B | B | -0.26 |
| *CCDC88C* | 14 | 91739387 | G | T | NM_001080414.4 | p.Pro1890His | c.5669C>A | 4.47E-05 | U | D | 5.13 |
| *CD74* | 5 | 149792207 | G | A | NM_001025159.2 | p.Arg36Cys | c.106C>T | 3.99E-06 | U |  | 4.82 |
| *CELA2B* | 1 | 15813856 | C | T | NM_015849.2 | p.Ser239Leu | c.716C>T | 0.000135 | U | D | 4.77 |
| *CENPS-CORT* | 1 | 10494710 | T | G | NM_198544.3 | | c.176-4T>G | 0.000335 |  |  |  |
| *CEP131* | 17 | 79173521 | G | A | NM_014984.4 | p.Gln341* | c.1021C>T | N/A |  |  | 3.67 |
| *CHRNB3* | 8 | 42587410 | TCAC | T | NM_000749.4 | p.His322del | c.964_966delCAC | 4.05E-06 |  |  |  |
| *CNDP1* | 18 | 72228253 | G | A | NM_032649.6 | p.Gly156Arg | c.466G>A | 8.78E-05 | U | U | 4.48 |
| *COL18A1* | 21 | 46876562 | G | C | NM_130444.3 | p.Trp373Ser | c.1118G>C | 0.000461 | D |  | 5.16 |
| *CORO7-PAM16* | 16 | 4457690 | A | G | NM_001201479.1 | | c.304-5T>C | 7.18E-05 |  |  |  |
| *CST2* | 20 | 23807139 | G | C | NM_001322.3 | p.Ser53Arg | c.159C>G | 7.95E-06 | B | B | 0.814 |
| *CYP27B1* | 12 | 58159000 | C | T | NM_000785.4 | | c.590-6G>A | 6.68E-05 |  |  |  |
| *DAGLB* | 7 | 6449833 | G | C | NM_139179.4 | p.Ser583Cys | c.1748C>G | 0.000521 | U | D | 5.94 |
| *DCAF4* | 14 | 73406542 | A | T | NM_001352449.1 | p.Asp42Val | c.125A>T | 4.82E-05 | U | U | 2.52 |
| *DCAF5* | 14 | 69589012 | T | C | NM_003861.2 | p.Ile94Val | c.280A>G | 0.000287 | B | B | 1.96 |
| *DENND1A* | 9 | 126146148 | C | A | NM_001352964.1 | p.Gly602Val | c.1805G>T | N/A | U |  | 3.32 |
| *DHRS12* | 13 | 52343408 | C | G | NM_001270424.1 | p.Gly243Ala | c.728G>C | 8.22E-05 | U | B | 2.75 |
| *DLG2* | 11 | 84028128 | C | T | NM_001206769.1 | p.Val21Ile | c.61G>A | 9.95E-05 | U | B | 4.8 |
| *DNAH14* | 1 | 225226436 | A | C | NM_001367479.1 | p.Asn467His | c.1399A>C | N/A | U |  | 2.7 |
| *DNM3* | 1 | 172007565 | C | A | NM_015569.5 | p.Pro319Gln | c.956C>A | 0.001105 | U | D | 6.03 |
| *DPF2* | 11 | 65101392 | G | A | NM_006268.5 | | c.32+3G>A | 0.000168 |  |  |  |
| *EAPP* | 14 | 34993984 | C | T | NM_018453.4 | p.Arg167His | c.500G>A | 0.000277 | B | B | -1.44 |
| *EFCC1* | 3 | 128753081 | T | C | NM_024768.2 | p.Leu453Pro | c.1358T>C | N/A |  |  | 0.313 |
| *ENTPD5* | 14 | 74436822 | T | C | NM_001321988.1 | p.Asp364Gly | c.1091A>G | N/A | B |  | 5.53 |
| *EPHA5* | 4 | 66270167 | A | T | NM_004439.7 | p.Ile572Asn | c.1715T>A | 0.000108 | U | U | 5.05 |
| *ERO1B* | 1 | 236389742 | C | G | NM_019891.3 | p.Lys293Asn | c.879G>C | 7.57E-05 | B | U | -3.19 |
| *F5* | 1 | 169492584 | T | C | NM_000130.4 | p.Met1967Val | c.5899A>G | 0 | U |  | 0.341 |
| *FAM200A* | 7 | 99145058 | C | T | NM_145111.4 | p.Val325Ile | c.973G>A | 0.000176 | B | B | -0.62 |
| *FAM207A* | 21 | 46363698 | GTCAGGAGAGGTGAGGCAGGCTCGAGTGCACGGAGCGTCCCTTCCA | G | NM_058190.4 | p.Ala86_Gly95del | c.254_283+15delGTGCACGGAGCGTCCCTTCCATCAGGAGAGGTGAGGCAGGCTCGA | 0.000549 |  |  |  |
| *FBRSL1* | 12 | 133160042 | A | C | NM_001142641.2 | p.Glu939Ala | c.2816A>C | N/A | U |  | 2.36 |
| *FLG2* | 1 | 15232935 | A | C | NM_001014342.2 | p.Phe303Val | c.907T>G | N/A | U |  | 3.86 |
| *GDF11* | 12 | 56142639 | G | A | NM_005811.4 | p.Asp239Asn | c.715G>A | 8.04E-06 | U |  | 3.94 |
| *GKAP1* | 9 | 86399761 | G | A | NM_025211.4 | | c.439-8C>T | 7.27E-05 |  |  |  |
| *GNE* | 9 | 36234070 | G | A | NM_005476.6 | p.Arg277Cys | c.829C>T | 1.99E-05 | U | U | 5.77 |
| *GSE1* | 16 | 85701766 | G | A | NM_014615.5 | p.Ala1051Thr | c.3151G>A | 4.16E-06 | B |  | 3.53 |
| *HAUS8* | 19 | 17163668 | T | C | NM_033417.2 | p.Asp299Gly | c.896A>G | 3.58E-05 | B | B | 2.28 |
| *HEXD* | 17 | 80398983 | C | G | NM_173620.2 | p.Leu365Val | c.1093C>G | 0.000485 | U | B | -4.49 |
| *HOMEZ* | 14 | 23744824 | TCTTCC | T | NM_020834.2 | p.Glu537fs | c.1608_1612delGGAAG | 0.00091 |  |  |  |
| *HOMEZ* | 14 | 23744822 | CA | C | NM_020834.2 | p.Asp538fs | c.1614delT | 0.000974 |  |  |  |
| *ITGA10* | 1 | 145534905 | C | G | NM_003637.5 | p.Ser603Cys | c.1808C>G | N/A |  |  |  |
| *KHDC4* | 1 | 155899090 | G | C | NM_014949.4 | p.Pro154Arg | c.461C>G | N/A | U |  | 5.45 |
| *KIF19* | 17 | 72346710 | G | A | NM_153209.4 | p.Ala462Thr | c.1384G>A | 4.02E-06 | B | U | 5.67 |
| *KRR1* | 12 | 75900396 | C | CAA | NM_007043.7 | | c.394-9_394-8dupTT | 0 |  |  |  |
| *LRP3* | 19 | 33695534 | C | A | NM_002333.4 | | c.261-10C>A | 0.000108 |  |  |  |
| *MAGI1* | 3 | 66023803 | C | T | NM_001033057.1 | p.Gly61Ser | c.181G>A | 6.60E-05 | B | B | 4.26 |
| *MANSC4* | 12 | 27916285 | G | T | NM_001146221.2 | p.Leu137Ile | c.409C>A | 0.000546 | B | U | 4.78 |
| *MAST3* | 19 | 18255302 | C | T | NM_015016.2 | p.Arg842Cys | c.2524C>T | N/A | U |  | 4.71 |
| *MEGF10* | 5 | 126781361 | G | T | NM_001256545.2 | p.Val902Phe | c.2704G>T | 0.000131 | U | B | 5.68 |
| *MLLT6* | 17 | 36872622 | T | C | NM_005937.3 | p.Ser347Pro | c.1039T>C | N/A |  |  | 4.25 |
| *MRAP* | 21 | 33686939 | C | A | NM_206898.1 | p.Ala95Asp | c.284C>A | 3.21E-05 | U | U | -3.45 |
| *MS4A7* | 11 | 60150732 | G | A | NM_021201.5 | p.Gly40Arg | c.118G>A | 8.36E-05 | D | U | -1.81 |
| *MUC17* | 7 | 100680542 | A | G | NM_001040105.1 | p.Thr1949Ala | c.5845A>G | 4.01E-05 | B | B | -0.62 |
| *MUC5AC* | 11 | 1220913 | C | T | NM_001304359.2 | | c.4990-8C>T | 0.000587 |  |  |  |
| *MYO18B* | 22 | 26286724 | T | C | NM_032608.6 | p.Ile1439Thr | c.4316T>C | 6.42E-05 | U | U | 5.64 |
| *MYO7A* | 11 | 76905558 | G | A | NM_000260.4 | p.Ala1438Thr | c.4312G>A | 8.57E-05 | U | B | 4.85 |
| *NADSYN1* | 11 | 71193973 | G | A | NM_018161.4 | p.Gly410Glu | c.1229G>A | 4.78E-05 | B | U | 4.06 |
| *NDUFB7* | 19 | 14677671 | G | C | NM_004146.6 | p.Leu63Val | c.187C>G | 0.000564 | U | U | 4.76 |
| *NEB* | 2 | 152496907 | C | T | NM_001271208.2 | p.Val2883Ile | c.8647G>A | 8.43E-05 | B | U | 6.07 |
| *NMT1* | 17 | 43159126 | A | G | NM_021079.5 | | c.240+6A>G | N/A |  |  |  |
| *NOS3* | 7 | 150697652 | G | A | NM_000603.5 | p.Val400Met | c.1198G>A | 0.000112 | D | D | 5.35 |
| *OR2F1* | 7 | 143657983 | A | C | NM_012369.2 | p.Lys307Thr | c.920A>C | N/A |  |  | 4.24 |
| *OR51J1* | 11 | 5424534 | G | C | NM_001348224.1 | p.Arg236Ser | c.708G>C | 0.000523 |  | U |  |
| *PAQR6* | 1 | 156214942 | G | T | NM_001272106.1 | p.Ser67Tyr | c.200C>A | 0.000219 |  |  | -1.48 |
| *PARP16* | 15 | 65578614 | C | T | NM_017851.5 | p.Asp51Asn | c.151G>A | 0.000104 | U | B | 3.29 |
| *PEX14* | 1 | 10689823 | G | A | NM_004565.3 | p.Val305Ile | c.913G>A | 9.88E-06 | B | U | 5.03 |
| *PIEZO1* | 16 | 88782329 | CCACT | C | NM_001142864.4 | | c.7316+8_7316+11delAGTG | 0.000263 |  |  |  |
| *PKD1* | 16 | 2185564 | G | C | NM_001009944.2 | p.Pro43Ala | c.127C>G | N/A | D |  | 3.03 |
| *PLEKHO1* | 1 | 150129619 | G | A | NM_016274.6 | p.Arg155Gln | c.464G>A | 0 | U | D | 4.69 |
| *PLOD1* | 1 | 12016980 | T | C | NM_000302.4 | p.Val217Ala | c.650T>C | 2.39E-05 | D | U | 3.09 |
| *PRKDC* | 8 | 48792125 | T | C | NM_006904.6 | p.His1720Arg | c.5159A>G | 3.60E-05 |  | U | 3.5 |
| *PRPF40B* | 12 | 50028379 | G | A | NM_001031698.2 | p.Arg332His | c.995G>A | 1.19E-05 | U | D | 4.16 |
| *PRR35* | 16 | 614855 | C | G | NM_145270.3 | p.Leu422Val | c.1264C>G | 1.16E-05 | U | D | 4.18 |
| *PRRC2C* | 1 | 171509282 | A | G | NM_015172.3 | p.Asn891Asp | c.2671A>G | 0.000223 | B |  | 4.57 |
| *PRSS3* | 9 | 33796772 | G | A | NM_007343.3 | p.Val115Met | c.343G>A | 4.38E-05 | D | D | 3.21 |
| *PRSS33* | 16 | 2835039 | A | T | NM_152891.2 | p.Cys216* | c.648T>A | 4.44E-05 |  |  | -4.39 |
| *R3HDM2* | 12 | 57648749 | TC | T | NM_001351206.1 | p.Asp979fs | c.2935delG | N/A |  |  |  |
| *RAB11FIP4* | 17 | 29850523 | C | A | NM_032932.6 | | c.930-7C>A | 7.96E-06 |  |  |  |
| *RFNG* | 17 | 80009501 | A | C | NM_002917.2 | p.Ser48Ala | c.142T>G | N/A | B |  | -5.67 |
| *RNF115* | 1 | 145688100 | T | A | NM_014455.4 | p.Cys265* | c.795T>A | N/A |  |  |  |
| *RRBP1* | 20 | 17602097 | G | C | NM_001365613.1 | | c.3414+6C>G | 0.000394 |  |  |  |
| *RTKN2* | 10 | 63995998 | C | G | NM_145307.4 | p.Gln171His | c.513G>C | N/A | U |  | 2.71 |
| *RTRAF* | 14 | 52465236 | C | T | NM_016039.3 | p.Pro104Leu | c.311C>T | 0.00144 | U | U | 5.14 |
| *SCN4A* | 17 | 62020273 | C | T | NM_000334.4 | p.Val1401Met | c.4201G>A | 0.000247 | U | B | 0.769 |
| *SCT* | 11 | 626959 | GGTGAACGTCCCGTCTGA | G | NM_021920.4 | p.Ser29fs | c.85_101delTCAGACGGGACGTTCAC | N/A |  |  |  |
| *SIGLEC1* | 20 | 3684079 | G | T | NM_023068.4 | p.Ser331Arg | c.993C>A | 0.000313 | U | U | 3.53 |
| *SLC16A3* | 17 | 80196722 | C | T | NM_001042422.2 | p.Ala423Val | c.1268C>T | 8.81E-05 | B | B | 1.83 |
| *SLC18A3* | 10 | 50818994 | G | A | NM_003055.3 | p.Gly70Ser | c.208G>A | 0.000299 | B | B | 2.03 |
| *SLC26A4* | 7 | 107330614 | T | C | NM_000441.1 | p.Ser399Pro | c.1195T>C | 0.000131 | U | D | 5.62 |
| *SLC26A4* | 7 | 107314609 | G | T | NM_000441.1 | p.Gly139Val | c.416G>T | 1.99E-05 | D | D | 5.29 |
| *SLX4* | 16 | 3640005 | C | T | NM_032444.4 | p.Ala1212Thr | c.3634G>A | 0.000322 | B | U | 0.489 |
| *SMOX* | 20 | 4164207 | G | A | NM_175839.3 | p.Arg479His | c.1436G>A | 3.98E-06 | U | D | 5.22 |
| *SVEP1* | 9 | 113169736 | A | G | NM_153366.4 | p.Ile2715Thr | c.8144T>C | 0.000149 |  | U | 4.73 |
| *TAF5L* | 1 | 229738105 | A | G | NM_014409.3 | p.Leu270Pro | c.809T>C | 7.96E-06 | B | B | 4.65 |
| *TBC1D16* | 17 | 77921640 | G | A | NM_019020.4 | | c.1542-10C>T | 0.000187 |  |  |  |
| *TCN1* | 11 | 59623518 | G | T | NM_001062.4 | p.Ser254* | c.761C>A | 6.01E-05 |  |  | 3.45 |
| *TEKT3* | 17 | 15234607 | C | A | NM_031898.3 | p.Trp99Leu | c.296G>T | 1.19E-05 | D | D | 5.4 |
| *TEP1* | 14 | 20836715 | C | T | NM_007110.5 | p.Gly2589Ser | c.7765G>A | N/A | D |  | 5.81 |
| *TH* | 11 | 2185524 | G | A | NM_000360.3 | p.Ser478Phe | c.1433C>T | N/A | B |  | 4.29 |
| *THADA* | 2 | 43779370 | G | A | NM_022065.4 | p.Thr928Ile | c.2783C>T | N/A | B |  | 4.67 |
| *TMEM74* | 8 | 109796488 | G | C | NM_153015.3 | p.Phe280Leu | c.840C>G | 0.000135 | B | U | 3.2 |
| *TRIM37* | 17 | 57140009 | G | C | NM_015294.6 | p.Ser287Arg | c.861C>G | 5.57E-05 | U | D | 1.19 |
| *TTLL10* | 1 | 1132810 | C | T | NM_001130045.1 | | c.1613-8C>T | 0.000217 |  |  |  |
| *TXLNA* | 1 | 32645914 | G | A | NM_175852.4 | | c.-32-1G>A | N/A |  |  |  |
| *UBN1* | 16 | 4908663 | C | G | NM_001079514.2 | p.Ala144Gly | c.431C>G | N/A | U |  | 5.63 |
| *UBR2* | 6 | 42600579 | T | G | NM_015255.2 | p.Asp494Glu | c.1482T>G | 0.000147 | B | B | 1.13 |
| *ULK1* | 12 | 132405847 | A | G | NM_003565.3 | | c.3098-4A>G | 4.05E-06 |  |  |  |
| *VPS13A* | 9 | 79829301 | T | C | NM_033305.3 | p.Phe251Phe | c.753T>C | 7.96E-06 |  |  |  |
| *XRN1* | 3 | 142031516 | T | G | NM_019001.4 | p.His1581Pro | c.4742A>C | N/A | B |  | -0.19 |
| *ZC3H18* | 16 | 88696980 | C | T | NM_001294340.1 | p.Pro909Leu | c.2726C>T | 0.000353 | U | U | 2.13 |
| *ZNF207* | 17 | 30694990 | G | C | NM_001098507.1 | p.Ser374Thr | c.1121G>C | N/A | U |  | 5.91 |
| *ZNF407* | 18 | 72775411 | A | G | NM_017757.2 | p.Ile1912Val | c.5734A>G | 4.31E-06 | U |  | 0.913 |
| *ZNF595* | 4 | 86510 | A | G | NM_182524.4 | p.Asn371Ser | c.1112A>G | 0.000775 |  |  |  |
| *ZNF613* | 19 | 52448669 | G | GT | NM_001031721.4 | p.Tyr512fs | c.1534dupT | 5.17E-05 |  |  |  |

Genomic coordinates are provided according to GRCh37/hg19 genome assembly. AA=Amino acid, AF=Allele frequency, U=Unknown, B=Benign, D=Deleterious or Damaging
